# High-altitude well log evaluation of a permafrost gas hydrate reservoir in the Muli area of Qinghai, China

**DOI:** 10.1038/s41598-018-30795-x

**Published:** 2018-08-22

**Authors:** Zhenzhou Lin, Heping Pan, Hui Fang, Wenli Gao, Dongming Liu

**Affiliations:** 1 Institute of Geophysics and Geomatics, China University of Geosciences (Wuhan), Wuhan, 430074 China; 2 Institute of Geophysical and Geochemical Exploration, CAGS, Langfang, 065000 China

## Abstract

The Muli area is the only permafrost region on the Chinese mainland wherein gas hydrates have been discovered. The gas hydrates are present in the fractures and pore spaces of the host rocks with a lamellar or micro-disseminated structure. By combining conventional and image logs, we describe the thickness of the permafrost layer and the well log responses of the gas hydrate reservoir, and calculate the porosity and gas hydrate saturation. We then analyze the advantages and disadvantages of different logging methods for evaluating gas hydrate reservoirs. Our results indicate that (1) gas hydrates are present below the permafrost in the Muli area, (2) gas hydrates predominantly occur in rock fractures, (3) the apparent resistivity is sensitive to gas hydrates present in pore spaces, and both apparent resistivity and acoustic logs are sensitive to gas hydrates present in fractures, (4) a density log is more appropriate for calculating porosity, and (5) gas hydrate saturation can be effectively calculated by the Archie equation, the modified Archie equation, and the Indonesian equation.

## Introduction

Gas hydrates are cage-structured compounds consisting of water and natural gas. They are mainly found in terrestrial permafrost regions and in marine sediments along the outer margins of continents, and they are a potential source of unconventional energy^1^. Permafrost-associated gas hydrates are primarily distributed in the high-latitude permafrost regions of the Arctic. These include the Prudhoe Bay-Kuparuk area on the North Slope of Alaska, USA, the Mackenzie Delta and Sverdrup Basin of Canada, the West-Siberian Basin, Lena-Tun-guska area, Timan-Pechora Basin, NE Siberia, and Kamchatka areas of Russia, the Svalbard Peninsula in Norway, and areas of Greenland^2–4^. At approximately 2.15 × 10^6^ km^2^, China has the third largest permafrost area in the world. Previous studies of permafrost regions in China have indicated that the Qiangtang Basin, Qilian montane area, Fenghuo-Wuli area, and the Mohe Basin may have experienced suitable conditions for gas hydrates to form. The gas hydrate resources in this region^4^ has been approximated to be 3.8 × 10^12^ m^3^. From 2008 to 2017, the China Geological Survey conducted a scientific drilling project to evaluate the presence of gas hydrates in the south of the Qilian Mountains in Qinghai, which included obtaining gas hydrate samples from boreholes. Meanwhile, several geological, geophysical, and geochemical surveys and laboratory tests have characterized the reservoir and confirmed several indicators of gas hydrates^5,6^. The geophysical surveys conducted in this area consisted of 2D seismic reflections, audio Magnetotellurics, and well logging^7^. This study focuses on the data from well logs, which were collected using a Micro-Logger II system (Robertson Geologging Ltd., UK). Well logging included the production of electrode resistivity logs, spontaneous potential logs, natural gamma rays, acoustic wave velocities, borehole temperatures, ultra-sonic image logs, and so on.

Gas hydrates form under particular temperature and pressure conditions, and they may decompose when the environment changes. Therefore, it is very difficult to obtain undisturbed samples of gas hydrates. Well logging may provide the *in situ* information of the downhole formation, and therefore the most adequate data on gas hydrate-bearing reservoirs. Most investigations of gas hydrate reservoirs use well logging methods^2,8–15^. In permafrost areas and marine sediments, the well log responses of gas hydrate reservoirs are characterized by high resistivity, high P-wave velocity, high neutron porosity, and low density^16–20^. The purpose of well logging evaluations is to transform well logging data into reservoir parameters, such as porosity and gas hydrate saturation, which are critical for the evaluation and production of gas hydrate resources.

Here, we first introduce a method of determining permafrost thickness using temperature logs. We then extract the log responses of the gas hydrate reservoir, and select tested models for computing the porosity and gas hydrate saturation of the reservoir^10,11,21–28^. Finally, we develop a set of well logging methods that can be used to evaluate high-altitude permafrost gas hydrate reservoirs.

## Geological Setting

**Figure 1:**
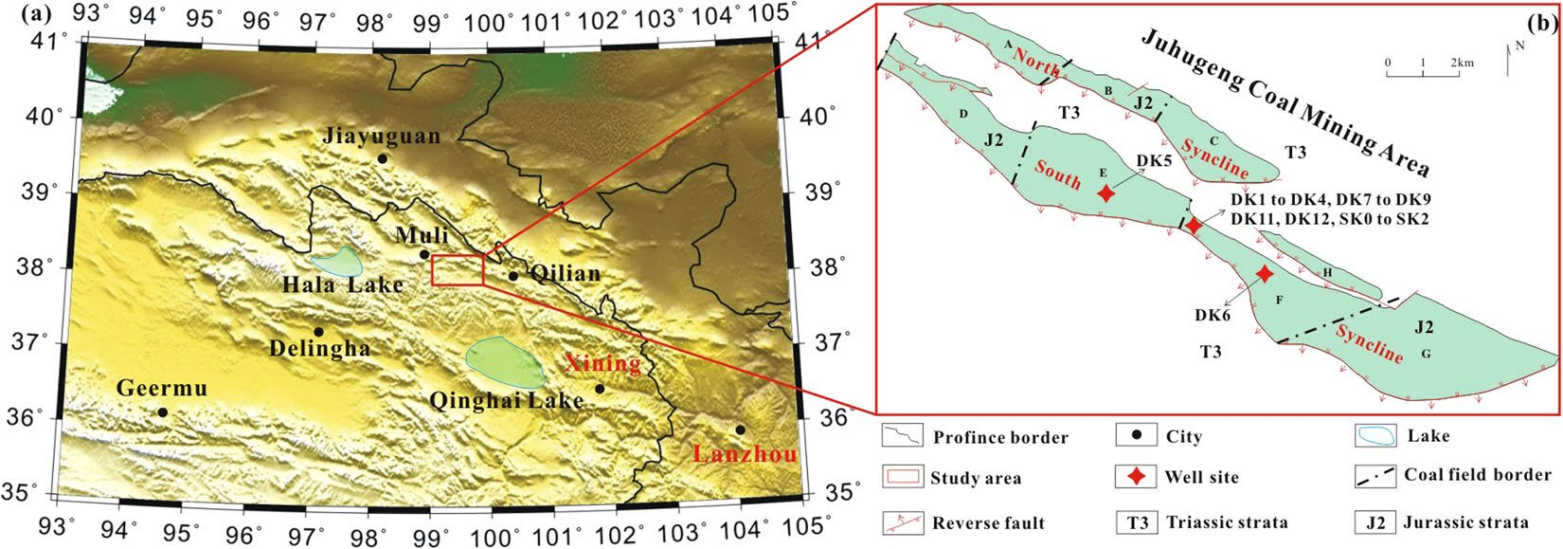
(**a**) Simplified geomorphological map of the study area (generated by free Generic Mapping tools v 5.4.3 software: http://gmt.soest.hawaii.edu/projects/gmt/wiki/Download). (**b**) Map of the Juhugeng coal mining area and the well site^29^ (generated by CorelDRAW ×7 software, Corel Corporation: http://www.corel.com/cn/).

The study area was located on the southern margin of the Qilian Mountain, at an elevation of 4100–4300 m and an annual mean surface temperature of 5.1 °C. The drill sites were located within the Juhugeng coal mining area of the Muli depression in the central Qilian Basin (Fig. 1). Previous geological studies have shown that the central part of the Juhugeng coal mining area is an anticline composed of Triassic strata, and the south and north flanks are two synclines composed of coal-bearing Jurassic strata^29,30^ (Fig. 1). At present, there are three coal fields located on the northern syncline and four coal fields on the southern syncline.

The formations discovered in the coal field belong to the Muli and Jiangcang groups. The lower member of the Muli Group was formed in a floodplain with a braided river, and is composed of a sequence of coarse siliciclastic rocks and basal conglomerate^29,30^. The upper member of the Muli Group was formed in lacustrine and marsh environments, and contains several mineable coal seams. The lower member of the Jiangcang Group was formed in deltaic and lacustrine environments, and contains two to six coal layers. The upper member of the Jiangcang Group was formed in a shallow to semi-deep lake environment, and consists of shales and siltstones. Thus, the study area encompasses several paleoenvironments that likely serve as rich sources of gas, coal-bed methane, hydrocarbon source rocks, and oil shales that could further provide abundant sources for the formation of gas hydrates^6^.

## Results and Discussion

### Permafrost thickness

**Figure 2:**
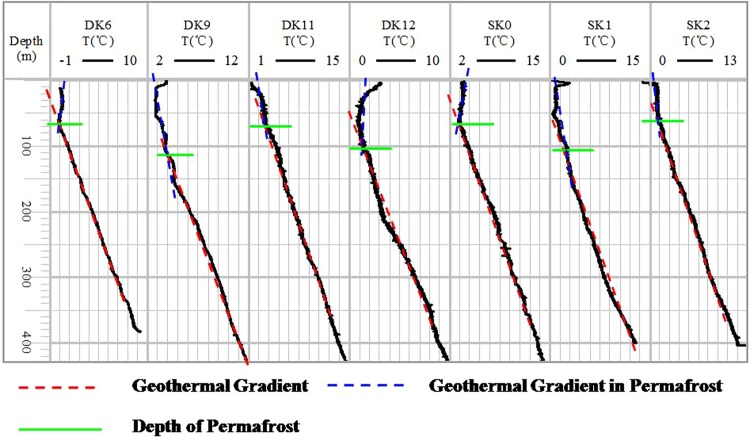
Temperature logs from seven boreholes (DK6, DK9, DK11–12, and SK0–2) in the Muli area.

Permafrost thickness is a key factor in the accumulation of gas hydrates, and it can determine whether the gas hydrate exists within the permafrost or below it. We can then use permafrost thickness to decide upon the appropriate reservoir model: (1) gas hydrate and ice or (2) gas hydrate and water. The minimum and maximum permafrost thicknesses in the Muli area were approximately 70 m and 120 m (Fig. 2), respectively. Gas hydrates occurred at depths of 130–400 m and were distributed discontinuously across this range^30^.

Temperature logs indicated that there were two dominant patterns of temperature with depth (Fig. 2). One such pattern was the “C” curve, in which temperatures were higher at the shallowest depths, then decreased, and finally increased to their highest values with increasing depth (e.g., DK12). The other pattern observed was the “L” curve, in which temperatures were steady in the top section and then increased in the lower section (e.g., DK11 and SK2). Regardless of the pattern observed, all temperature logs could be used to determine the thickness of the permafrost region.

Drilling engineers must circulate mud to protect the coal mining area from collapse; however, temperature well logging requires this circulation to be halted for a long period of time so that there is no heat exchange between the mud and the rock units. In this study, temperature logs were generated 24–48 h after mud circulation had ceased; however, most wells exhibited a temperature above 0 °C. Thus, we suggest that when temperature logs cannot collect data at 0 °C, the resulting curve will display a “C” or “L” pattern. The intersection between the deep gradient lines of the host rock and the permafrost region can be considered as the bottom of the permafrost^2^.

### Well log responses of the gas hydrate reservoir

The Muli area satisfies the requirements for the generation of gas hydrates based upon the gas source and permafrost conditions^4–6^. The general pattern of gas hydrate accumulation is as follows: natural gas from the pyrolysis of petroliferous gas migrates up-section along faults, accumulating in shallow rock units due to the sealing of mudstone and oil shales, and forms gas hydrates when it combines with water in permafrost regions^31^. Moreover, gas hydrates occur in cracks in rocks with lamellar structures or in the pore spaces of rocks with micro-disseminated structures. The lithology of the gas hydrate reservoir in this study is predominantly mudstone, oil shale, siltstone, and fine sandstone. The occurrence of gas hydrates varied with lithology and structure, so it is reasonable to analyze the log responses of the reservoir according to its lithology.

Table 1 summarizes the well log responses of the gas hydrate reservoir in the Muli area. Geological and mud logging indicated the presence of 67 layers of known or suspected gas hydrate reservoir within the 10 study wells. The accumulated thickness of the reservoir was 241 m (Table 2). The accumulated thickness of the gas hydrate-bearing sandstone, mudstone, and shale reservoirs were 89.1 m, 46.5 m, and 105.4 m, respectively. Thus, gas hydrates in the study area occur predominantly in fractures within mudstone and shale as both lithologies are characterized by low porosity. We evaluated the well log responses of each lithology by computing the resistivity, velocity, and density anomalies for each of the 67 gas hydrate-bearing layers and comparing them to layers without gas hydrates.

**Table 1:** Well logging responses of the gas hydrate reservoirs in the Muli area.

Reservoir type	Lithology	Resistivity	P-wave velocity	Density
Pore type	Sandstone	Increase	Small anomaly	No anomaly
Fracture type	Mudstone	Increase	No anomaly	Decrease
	Shale	Increase	Significant increase	No anomaly

**Table 2:** Distribution of the gas hydrate reservoir identified by well logging.

Borehole ID	Medium sandstone		Fine sandstone		Siltstone		Mudstone		Shale		Total thickness (m)
	Layers	Thickness (m)	Layers	Thickness (m)	Layers	Thickness (m)	Layers	Thickness (m)	Layers	Thickness (m)	
DK1	/	/	1	3.4	5	10.6	/	/	/	/	14.0
DK2	2	4.6	2	2.8	/	/	1	2.7	7	61.2	71.3
DK3	1	1.8	/	/	/	/	6	17.9	4	9.2	28.9
DK6	/	/	1	4.5	/	/	1	2.5	/	/	7.0
DK8	1	2.6	1	2.9	4	16.8	5	8.6	6	19.3	50.2
DK9	2	6.9	1	2.0	3	15.6	1	6.8	3	9.0	40.3
DK12	/	/	/	/	/	/	1	2.8	/	/	2.8
SK0	1	1.5	/	/	/	/	1	5.2	/	/	6.7
SK1	/	/	/	/	/	/	/	/	2	6.7	6.7
SK2	1	4.3	/	/	3	8.8	/	/	/	/	13.1
Total	8	21.7	6	15.6	15	51.8	16	46.5	22	105.4	241

#### Pore-type gas hydrate reservoir (sandstone)

**Figure 3:**
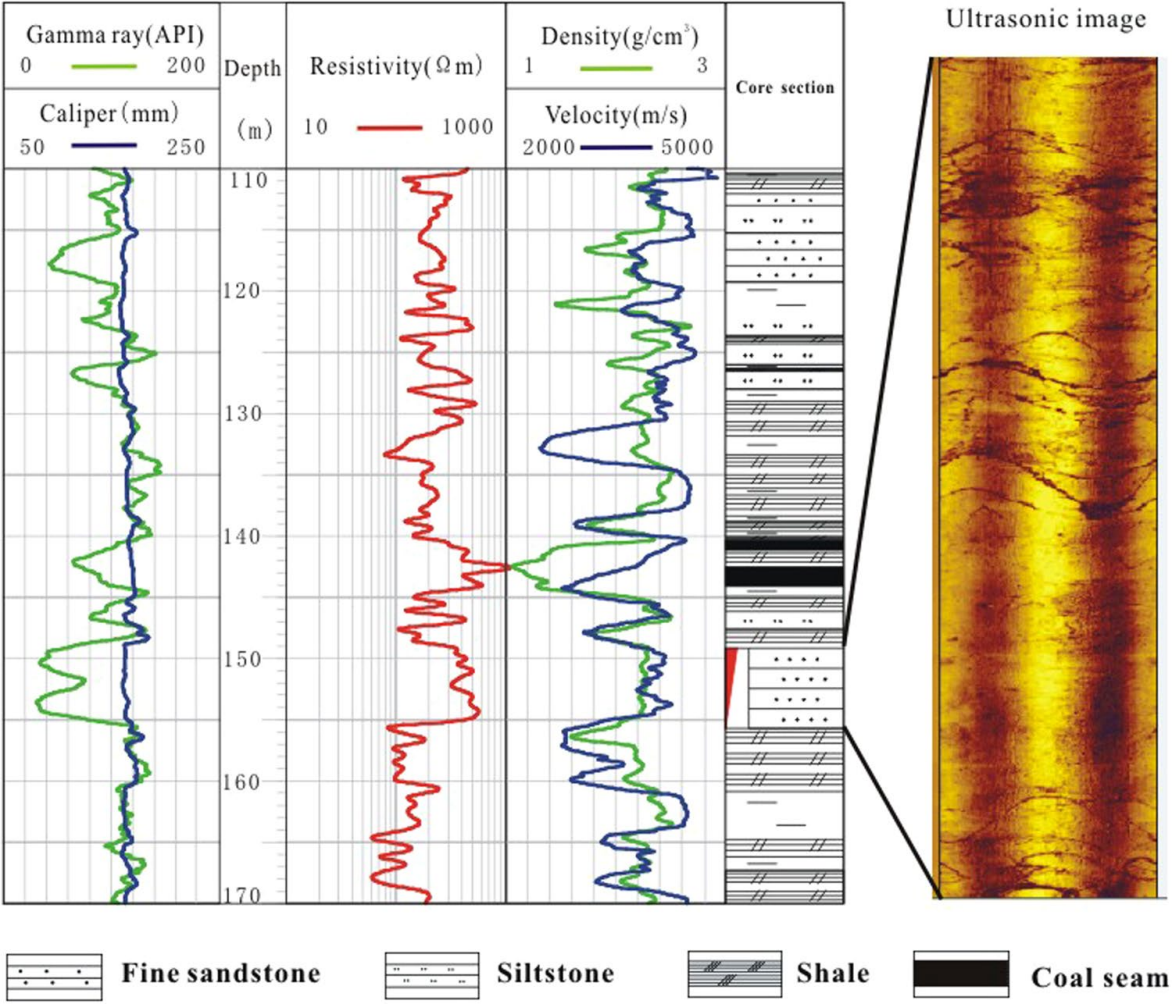
Well logs of sandstone (borehole DK8) with and without gas hydrate (red triangle indicates the gas hydrate layer).

The pore-space in the sandstone provides storage for gas hydrates. Well DK8 (fine sandstone) penetrated a gas hydrate reservoir from 150–155 m and exhibited a 200 Ωm increase in resistivity, a 150 m/s increase in velocity, a 0.05 g/cm^3^ decrease in density, and a 5 mm increase in borehole diameter compared to the non-gas hydrate-bearing layers from 116–119 m (Fig. 3). The ultrasonic image of this section is bright and displays few dark cracks, indicating that the sandstone of is relatively intact with few fractures.

**Figure 4:**
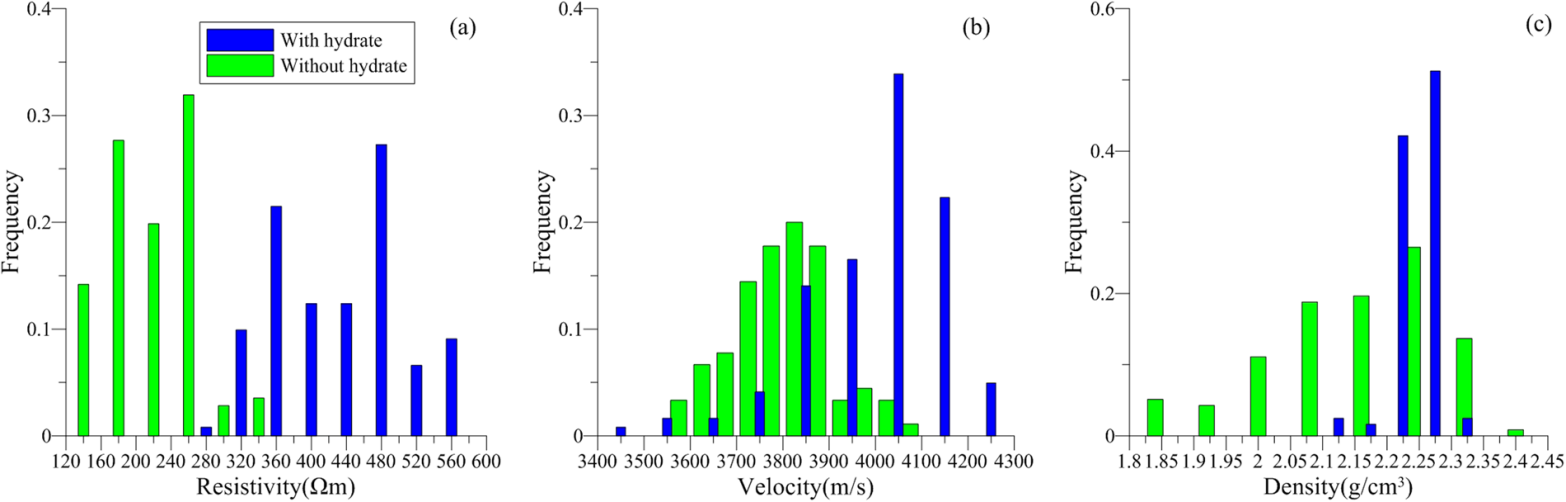
(**a**) Resistivity (Ωm), (**b**) velocity (m/s), and (**c**) density (g/m^3^) responses of sandstone reservoirs with (blue bars) and without (green bars) gas hydrate in the study area.

Twenty-nine gas hydrate-bearing sandstone formations were identified in the boreholes of the study area. Gas hydrate-bearing sandstones had resistivities of 370–490 Ωm, P-wave velocities of 3800–4100 m/s, and densities of 2.2–2.3 g/cm^3^ (Fig. 4). The formation of gas hydrates also generates a ‘salt discharge’ effect that can lead to a decrease in local conductivity. Thus, the apparent resistivity of the gas hydrate-bearing sandstone was twice that of a sandstone saturated with water. However, the apparent resistivity of the gas hydrate reservoir marine and polar permafrost areas are as much as 50 times that of a sandstone saturated with water^32^. This may be attributed to the low concentration of gas hydrates in the Muli sandstones.

In marine sediments, the velocities of gas hydrate reservoirs are important because they predominantly occur in uncemented rock units, and the generation of gas hydrates can change the physical properties of the reservoir and further alter rock velocities and densities. However, in the Muli area, gas hydrates occur in the sandstone that has already undergone diagenesis, so the generation of gas hydrates should not substantively alter the physical properties of the rock. This explains the lack of velocity and density anomalies in our results (Fig. 4).

#### Fracture-type gas hydrate reservoir (mudstone and shale)

**Figure 5:**
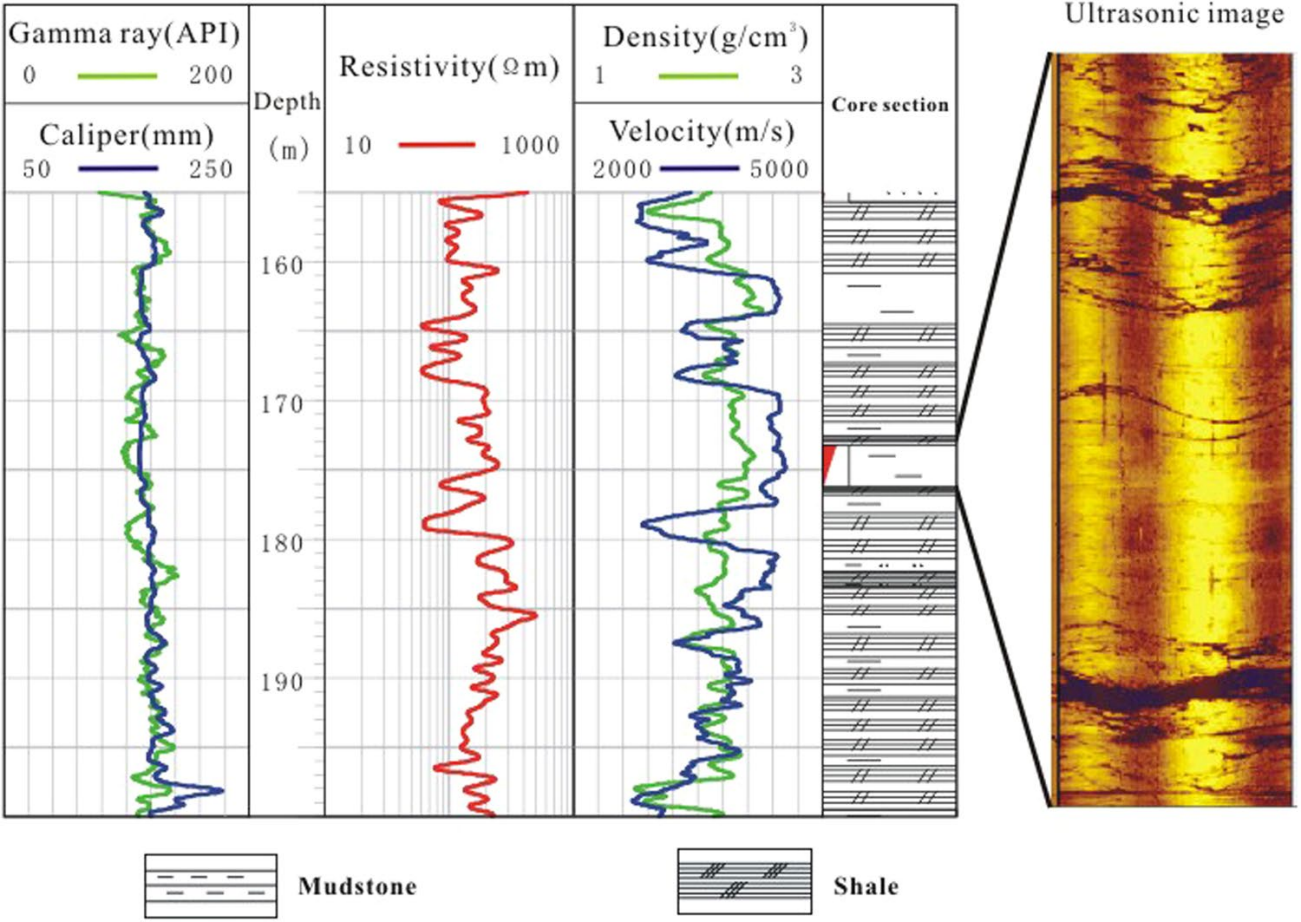
Well logs of the mudstone (borehole DK8) with and without gas hydrate (red triangle indicates the gas hydrate layer).

A mudstone gas hydrate reservoir was observed in well DK8 at a depth of 173–175 m. The ultrasonic image logs of this section indicated that the rock was less broken with some developed fractures. Two wide fractures were observed that provided storage for gas hydrates. Compared to the mudstone reservoir without gas hydrates from 161–164 m, the gas hydrate-bearing mudstone exhibited a 100 Ωm increase in resistivity, a 50 m/s increase in velocity, a 0.2 g/cm^3^ decrease in density, and a 2 mm increase in borehole diameter (Fig. 5).

**Figure 6:**
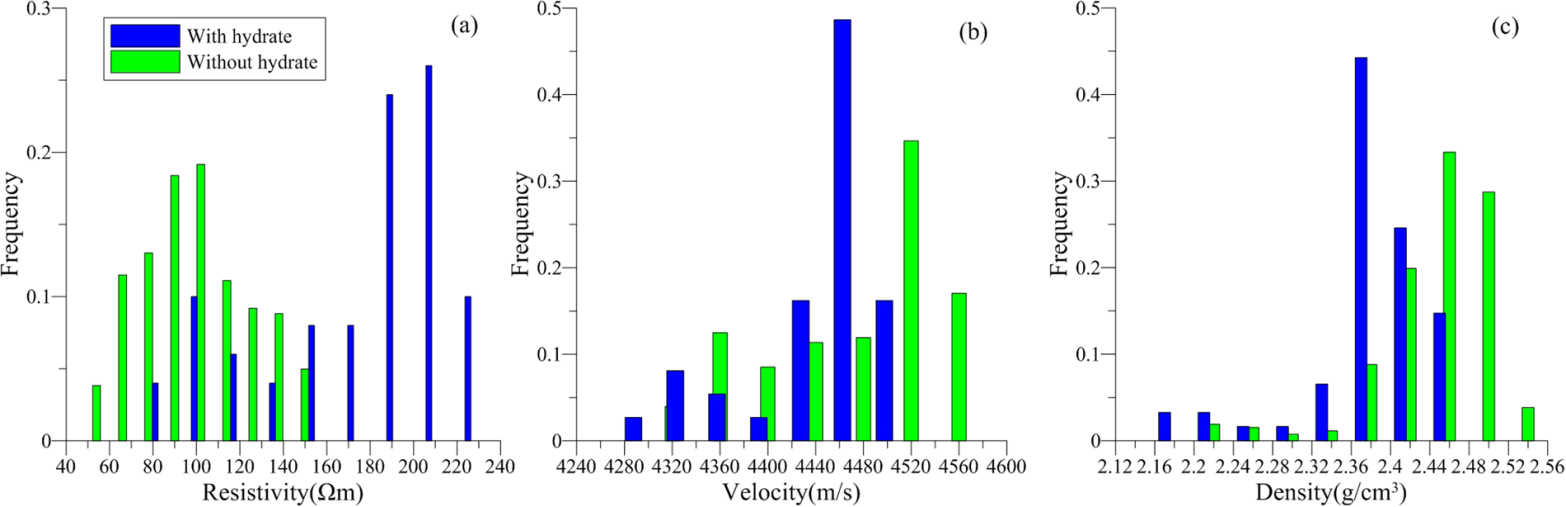
(**a**) Resistivity (Ωm), (**b**) velocity (m/s), and (**c**) density (g/m^3^) responses of mudstone reservoirs with (blue bars) and without (green bars) gas hydrate in the study area.

We identified 16 gas hydrate-bearing mudstone layers in the boreholes of study area. The gas hydrate-bearing mudstones had resistivities of 150–250 Ωm, P-wave velocities of 4400–4600 m/s, and densities of 2.36–2.44 g/cm^3^ (Fig. 6). Compared to the mudstone without gas hydrate, the resistivity of the gas hydrate-bearing mudstone was 2–3 times higher, the density was slightly lower, and the P-wave velocity showed no prominent variation. This indicates that the formation of gas hydrates in mudstone fractures affected some of the physical properties of the host rock.

**Figure 7:**
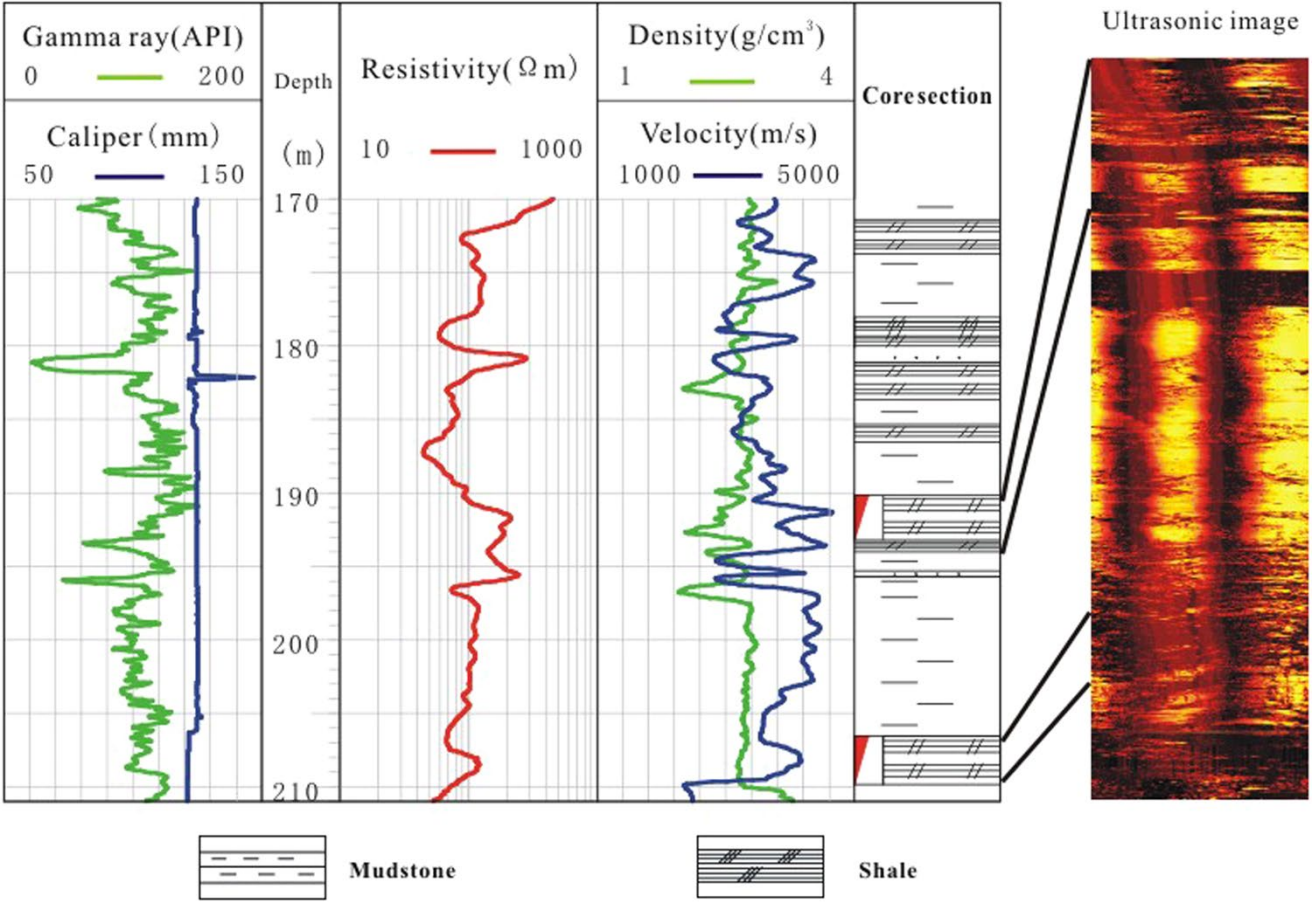
Well logs of the shale (borehole DK9) with and without gas hydrate (red triangle indicates the gas hydrate layer).

Two gas hydrate-bearing shale layers were found in well DK9 between 190 and 209.5 m depth, which also contained oil shale, mudstone, and fine sandstone. According to the ultrasonic logging images, cracks were well-developed in the shale formation, providing storage for gas hydrates (Fig. 7). Compared to shales without gas hydrates, the upper gas hydrate-bearing shale exhibited a 120 Ωm increase in resistivity, a 1800 m/s increase in velocity, a 0.2 g/cm^3^ decrease in density, and a 14 mm increase in borehole diameter. The lower gas hydrate-bearing shale revealed a 40 Ωm increase in resistivity, a 1500 m/s increase in velocity, a 0.01 g/cm^3^ decrease in density, and an 11 mm increase in borehole diameter.

**Figure 8:**
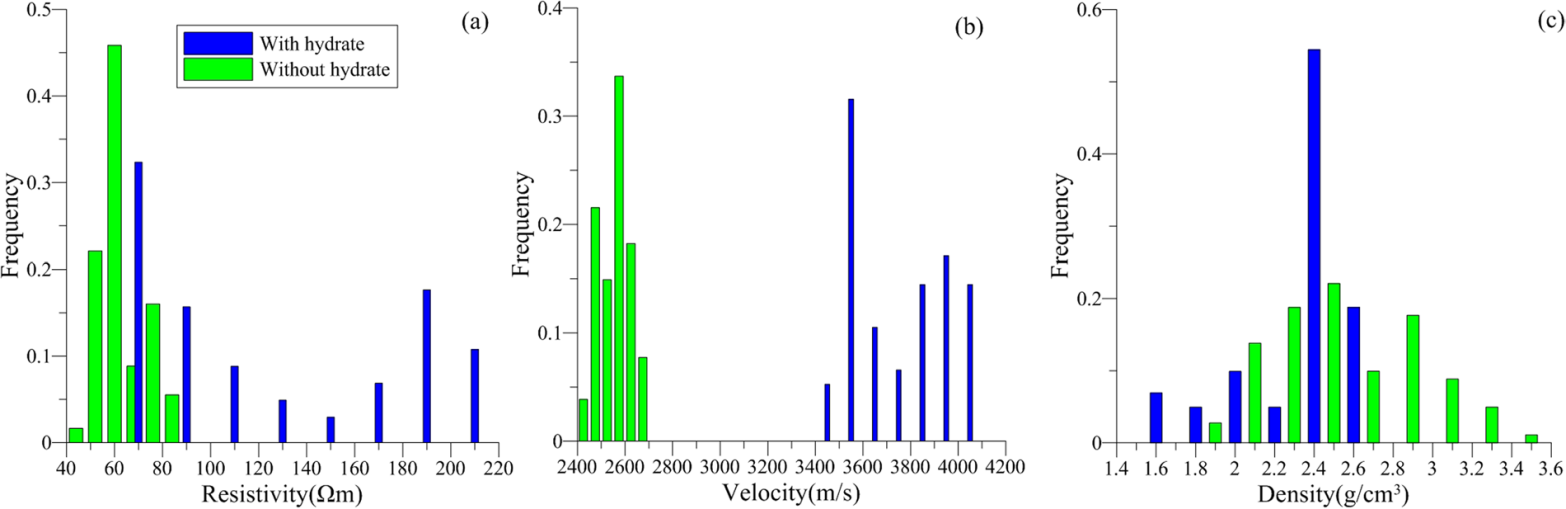
(**a**) Resistivity (Ωm), (**b**) velocity (m/s), and (**c**) density (g/m^3^) responses of shale reservoirs with (blue bars) and without (green bars) gas hydrate in the study area.

We identified 22 gas hydrate-bearing shale layers in the boreholes of study area. The gas hydrate-bearing shales had resistivities of 75–210 Ωm, P-wave velocities of 3600–4600 m/s, and densities of 2.4–2.6 g/cm^3^ (Fig. 8). Compared to shales without gas hydrates, the resistivity of the gas hydrate-bearing shale was much higher, as much as five times higher, the P-wave velocity was 1.5 times higher, and the density exhibited no obvious anomalies. The increase in P-wave velocities indicates that the formation of gas hydrates could enhance the consolidation effect of the rock. By integrating the well logging characteristics of the gas hydrate-bearing mudstone and shale, we conclude that fracture-type gas hydrate-bearing reservoirs exhibit clear resistivity anomalies. The greater the P-wave velocity anomaly, the more fractures and crushed zones exist in the gas hydrate-bearing rock unit.

#### Gas hydrate-bearing reservoir comparisons

Similar to well logs from another permafrost region, represented by the Northwest Eileen State-2 well, and the marine well from the Deep Sea Drilling Project (DSDP), Leg 84, Site 570 (Table 3), the resistivity logs increased in the gas hydrate-bearing units in the Muli area. However, unlike these other regions, the acoustic and density logs of pore-type gas hydrate-bearing reservoirs in the study area showed no anomalies. Conversely, the acoustic and density logs of fracture-type gas hydrate-bearing reservoirs show similar anomalies to these other regions. The caliper log was slightly increased in the Muli area; however, the anomalies were weaker due to the lithology and low volume of gas hydrates.

**Table 3:** Gas hydrate well log responses from a permafrost region (Northwest Eileen State-2 well) and the sea floor^2^ (DSDP Leg 84 Site 570 well).

Log data	Northwest Eileen State-2 well	DSDP Leg 84 Site 570 well
Acoustic transit-time log	Decrease	Responded to gas hydrates
Density	Decrease	Responded to gas hydrates
Dual induction log	Increase	Responded to gas hydrates
Caliper log	Increase	Responded to gas hydrates

### Well log evaluation

#### Porosity

**Figure 9:**
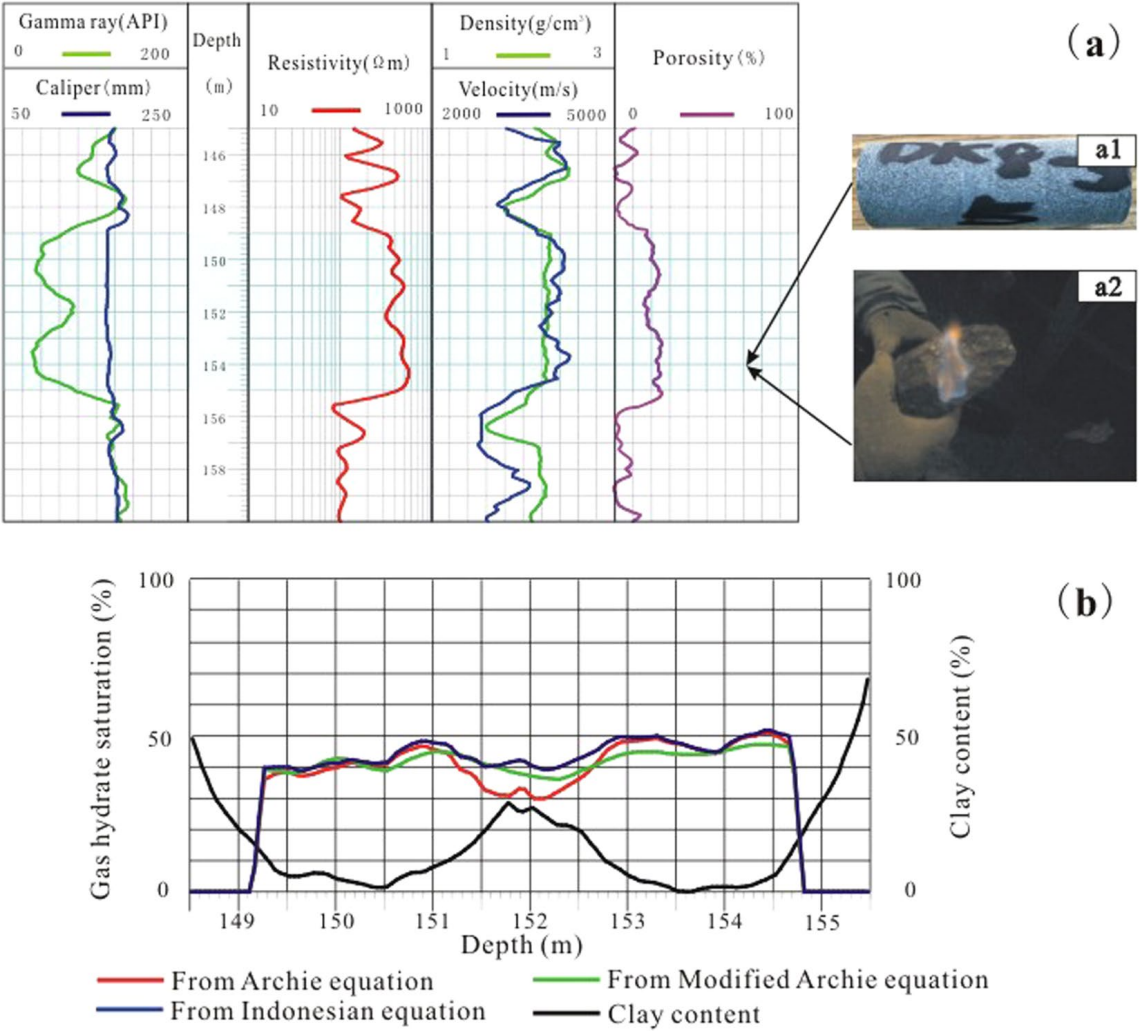
(**a**) Well logs from borehole DK8. (**b**) Gas hydrate saturations estimated from the four models employed.

The porosity of the Muli Group as calculated by core testing was 5–10%. Core test porosities yield the pore space occupied by water, including free water, interlayered water, and irreducible water. Most porosity well logs measure a parameter that reflects the water content of the unit, so the core test porosity and the porosity computed from well logs should be consistent. In this study, core porosity was measured by a gas expansion method. That the porosity calculated by the density log (ϕ_DEN_) is closer to the core test porosity than the porosity calculated by the acoustic log (ϕ_AC_) is shown in Table 4. Excluding the effect of borehole diameter, one possible reason for this pattern is that the acoustic log was affected by methane dissolved in the mud because gas hydrates can decompose into methane when the temperature and pressure conditions are altered by drilling. Thus, the density log should be used to compute the porosity of rocks when the gas hydrate content is high as it appears to be more robust to the environmental degradation of gas hydrates. Both density and porosity varied greatly among the boreholes and lithologies. Figure 9a indicates that the porosity calculated by the density log is ~20% and pore connectivity should be studied in the future.

**Table 4:** Core test-measured porosity (%) and well log-calculated porosity (% density and % acoustic).

Borehole ID	Depth (m)	Lithology	Core test porosity (%)	ϕ_DEN_ (%)	ϕ_AC_ (%)
DK2	108.50	medium sandstone	7.07	5.8	4.5
	110.50	medium sandstone	4.99	9.2	6.5
	115.50	mudstone	6.25	8.2	0.1
	387.50	coarse sandstone	5.47	8.6	1.5
	394.00	oil shale	8.19	8.2	0.1
	402.00	medium sandstone	3.81	6.5	0.1
	422.50	mudstone	4.90	4.4	2.2
	435.20	medium sandstone	5.56	8.4	2.0
DK3	69.76	siltstone	4.84	5.1	3.8
	87.00	coarse sandstone	9.40	6.2	12
	108.50	fine sandstone	7.07	3.0	7.5
	110.50	coarse sandstone	4.99	4.6	9.1
	113.00	coarse sandstone	6.03	3.8	10.7
	135.50	oil shale	3.21	3.2	7.2

#### Gas hydrate saturation

The gas hydrate saturation calculated by the empirical equation based on the resistivity log performed better than that based on the acoustic log; thus, resistivity logs are more suitable for the evaluation of gas hydrate saturation in the Muli area. Using resistivity logs, we adopted the Archie equation, the modified Archie equation, and the Indonesian equation to compute gas hydrate saturation.

Using the DK8 well as an example, core analysis at a depth of 154 m revealed a gas hydrate saturation of ~50%. Figure 9b illustrates the calculated gas hydrate saturation of the pore-type fine sandstone at a depth interval of 148–155 m, indicating that the values calculated by the Archie equation, the modified Archie equation, and the Indonesian equation were close to those derived from the core tests, and therefore suitable for evaluating gas hydrate saturation in the Muli area. Additionally, the Indonesian equation appeared to be suitable for units with low salinity waters, as exhibited in the study area, where resistivity of the formation water *R*_*w*_ = 3 Ωm. The modified Archie equation and the Indonesian equation can be used for units with high clay contents (V_sh_ ≥ 10%), because the equations contain correction terms to overcome the effect of clay.

As the drilling process can change the temperature and pressure conditions of the host rock and lead to decomposition of gas hydrates, both gas hydrate and gas are present in the pores, leading to a greater gas hydrate saturation when calculated by an acoustic log. Therefore, the acoustic log must be corrected before being used to compute gas hydrate saturation. The correction equation can be obtained by laboratory experiments.

The selection of a gas hydrate saturation model depends upon the distribution of gas hydrates and clay, pore type, lithology, and structure. While it remains a complex problem, it will plan an important role in the evaluation of gas hydrate reservoirs in the future. We tested three models in the Muli area and achieved reasonable results, which can provide insights into the evaluation of gas hydrate saturation in areas with similar geologic histories.

## Conclusions

The range of the permafrost thickness in the Muli area is from 70 to 120 m. The pore-type gas hydrate reservoir can be identified by apparent resistivity logs, while the fracture-type gas hydrate reservoir can be identified by apparent resistivity and acoustic logs. The gas hydrate-bearing rocks of the study area are predominantly fracture-type reservoirs, which exhibit lower apparent resistivity values (75–250 Ωm), similar P-wave velocities (3600–4600 m/s), and higher densities (2.36–2.6 g/cm^3^) than the pore-type reservoirs. The pore-type gas hydrate reservoir has an apparent resistivity of 370–490 Ωm, a P-wave velocity of 3800–4100 m/s, and a density of 2.2–2.3 g/cm^3^. Using the density log is a particularly effective method for calculating reservoir porosity in this area. Our study indicates that the Archie equation is suitable for evaluating the gas hydrate saturation in formations with low clay contents (V_sh_ < 10%), while the modified Archie equation and the Indonesian equation are both suitable for formations with high clay contents (V_sh_ ≥ 10%).

## Methods

### Determining permafrost thickness

The thickness of the permafrost was determined using temperature logs. Within these temperature logs, obvious variations in the geothermal gradient were observed, which indicated the bottom depth of the permafrost.

**Figure 10:**
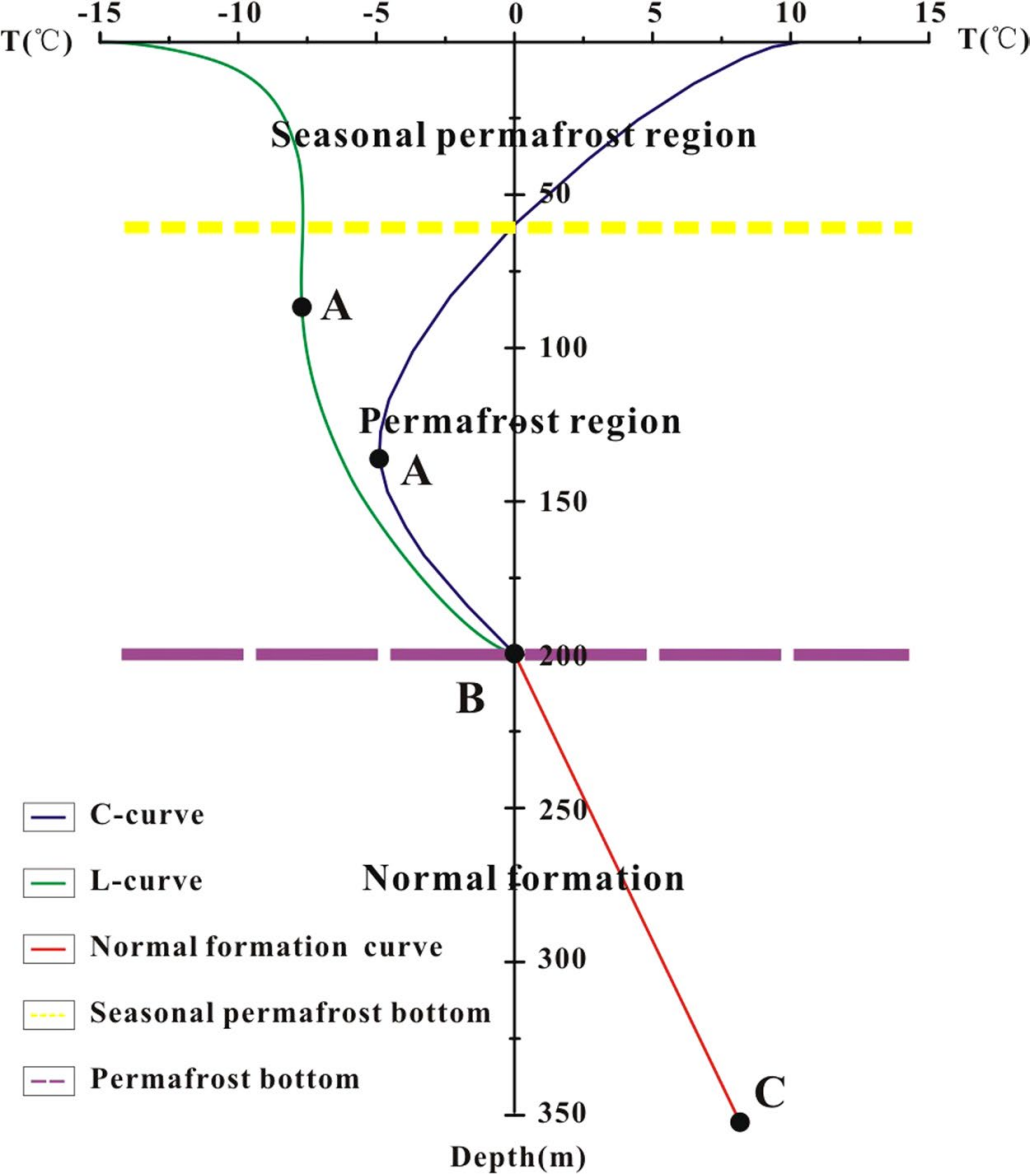
Schematic of the borehole temperature variation in the permafrost region.

We constructed a schematic model to illustrate the methods used to determine the bottom depth of the permafrost (Fig. 10). For a “C” temperature log curve, there were three components: the seasonal permafrost layer, the permafrost region, and the host rock. The thickness of the seasonal permafrost layer is affected by the seasons, wherein it is thinner in the summer and thicker in the winter. The minimum temperature appeared in the permafrost region. For a “L” temperature log curve, there was no seasonal permafrost layer. This pattern was observed in the borehole located on the lee side of a mountain at high altitude, so the surface temperature was below 0 °C even in the summer. However, the “C” temperature log curved may change to a “L” curve in the winter due to the decrease in surface temperatures.

The “C” and “L” temperature log curves overlapped below the B point shown in Fig. 10, indicating that the section below the B point is not affected by the permafrost region, and that the temperature logs in the lower section should exhibit a steady slope (Fig. 10). The slope of the section BC (Fig. 10) is close to the geothermal gradient in the study area. Point A represents the minimum value of the permafrost region. Therefore, the intersection point of lines AB and BC indicate the bottom depth of the permafrost region (Fig. 2).

### Calculation of porosity

Porosity is typically calculated by acoustic (AC) logs, density (DEN) logs, and neutron porosity (CNL) logs. Resistivity logs and nuclear magnetic resonance (NMR) logs are also used to compute the porosity of gas hydrate reservoirs, but porosities computed by resistivity logs may be lower than the actual porosities because the resistivity of gas hydrates is much higher than that of pore water^10,11,21–25^. A NMR log measures the porosity filled by free fluids; as gas hydrates are solid, it is not difficult to calculate the saturation of gas hydrates by combining other porosity logs and a NMR log^26^.

CNL and NMR have not been previously applied in the study area; therefore, in this study, density and acoustic logs were used to compute the porosity of gas hydrate reservoirs (Table 5) and compared to porosity determined by core tests.

**Table 5:** Equations used for calculating reservoir porosity.

Well logs	Equation	Parameter description
Density log	$\varphi =\frac{{\rho }_{{\rm{ma}}}-{\rho }_{{\rm{b}}}}{{\rho }_{{\rm{ma}}}-{\rho }_{{\rm{h}}}}-{V}_{{\rm{sh}}}\frac{{\rho }_{{\rm{ma}}}-{\rho }_{{\rm{sh}}}}{{\rho }_{{\rm{ma}}}-{\rho }_{{\rm{h}}}}$	ρ_ma_ = matrix density; ρ_b_ = density log; ρ_h_ = density of gas hydrate; ρ_sh_ = shale density; V_sh_ = clay content; ϕ = porosity.
Acoustic log	$\varphi =\frac{AC-A{C}_{ma}}{A{C}_{\varphi }-A{C}_{ma}}\cdot \frac{1}{{C}_{p}}-{V}_{sh}\frac{A{C}_{sh}-A{C}_{ma}}{A{C}_{\varphi }-A{C}_{ma}}$	AC = acoustic log; AC_ma_ = acoustic transit time of the rock matrix; AC_ϕ_ = acoustic transit time of the water; AC_sh_ = acoustic transit time of the shale; V_sh_ = clay content; ϕ = porosity.
Constants^32,33^	ρ_ma_ = 2.65 g/cm^3^; ρ_h_ = 0.9 g/cm^3^; ρ_sh_ = 2.3 g/cm^3^; AC_ma_ = 182 μs/m; AC_ϕ_ = 620 μs/m; AC_sh_ = 250 μs/m; Cp = 1.68–0.0002 × H (H is depth).	

### Calculation of gas hydrate saturation

The composition and structure of gas hydrate-bearing reservoirs are very complex; therefore, we simplified the model structure during calculation. Because of this simplification, it was prudent to use multiple models to improve the accuracy of our interpretations. As the gas hydrate-bearing reservoir was below the permafrost, gas hydrate saturation was evaluated as a function of the primary matrix (i.e., silicate, calcitic, or clay) and pore fluid (i.e., bond water, free water, or gas hydrate).

The evaluation of gas hydrate saturation typically employs resistivity and acoustic logs because the resistivity and acoustic properties of gas hydrate differ from the other components of the host rocks^10,11,21–25,27,28^. The different models used for evaluating gas hydrate saturation evaluation are listed in Table 6. We performed core tests in the laboratory and obtained the values of the scale factor (*a*), the cementation factors (*m* and *b*), and the saturation factor (*n*) of the study area. Due to the lack of local water analyses, we calculated the resistivity of the formation water (*R*_*w*_) using the apparent resistivity of the water saturated rock units (Eq. 1):

1 $$Rw=\frac{{\varphi }^{m}\,Rt}{ab}$$

where *ϕ* is porosity and *Rt* is rock unit resistivity. The resulting *R*_*w*_ of the study borehole is ~3 Ωm. To obtain the resistivity of the units saturated by water (*R*_*o*_), we used the following equation (Eq. 2):

2 $$Ro=\frac{aRw}{{\varphi }^{m}}$$

where *a*, *b*, *m*, and *n* were obtained by core tests and the other empirical parameters were from the literature.

**Table 6:** Models used for evaluating gas hydrate saturation.

Model	Model equation	Parameter description
Archie equation	${S}_{{\rm{h}}}=1-{(\frac{{\rm{a}}{R}_{{\rm{w}}}}{{\varphi }^{{\rm{m}}}{R}_{{\rm{t}}}})}^{1/n}$	*a* = scale factor; *m* = cementation factor; *n* = saturation factor; ϕ = porosity; R_t_ = resistivity of the formation; R_w_ = resistivity of the formation water; S_h_ = gas hydrate saturation
modified Archie equation	${S}_{h}={\rm{1}}-{(\frac{{R}_{0}}{{R}_{{\rm{t}}}})}^{\frac{1}{{\rm{n}}}}$	R_0_ = resistivity of the formation saturated by water
Indonesian equation	$\sqrt{{C}_{{\rm{t}}}}=\sqrt{\frac{{C}_{{\rm{w}}}}{F}}{{S}_{{\rm{w}}}}^{{\rm{n}}/2}+{{V}_{{\rm{sh}}}}^{1-{V}_{{\rm{sh}}}/2}\sqrt{{C}_{{\rm{sh}}}}{{S}_{{\rm{w}}}}^{{\rm{n}}/2}\,{S}_{h}=1-Sw$	C_t_ = conductivity of formation; C_w_ = conductivity of formation water; C_sh_ = conductivity of shale; V_sh_ = clay content; S_w_ = water saturation
Constants^32,33^	a = 0.91; m = 2.1; b = 0.98; n = 3.2; R_w_ = 3 Ωm; V_w_ = 1.5 km/s; V_h_ = 3.35 km/s; V_ma_ = 5.4 km/s.	

## References

[1] Shi, D. *et al*. Research Progress in Gas Hydrate Abroad. Lanzhou University: China Lanzhou (1992).

[2] Collett, T. S. Well log evaluation of natural gas hydrates. U. S. Geological Survey: Colorado (1992).

[3] Zheng Rong C (2016). Review of natural gas hydrates as an energy resource: Prospects and challenges. Applied Energy..

[4] Zhu YH (2011). Resource potential and reservoir distribution of natural gas hydrate in permafrost areas of China. Natural Gas Industy..

[5] Zhu YH (2009). Gas Hydrates in the Qilian Mountain Permafrost, Qinghai, Northwest China. Acta Geologica Sinica..

[6] Lu ZQ (2010). Basic geological characteristics of gas hydrates in Qilian Mountain permafrost area, Qinghai Province. Mineral Deposits..

[7] Fang H (2017). Main achievements of gas hydrate exploration technology in permafrost regions of China. Geophysical & Geochemical Exploration..

[8] Collett, T. S. & Lee, M. W. Downhole well log characterization of gas hydrates in nature-a review. The 7th International Conference on Gas Hydrates: United Kingdom (2011).

[9] Collett, T. S. & Lee, M. W. Well Log Characterization of natural Gas-Hydrates. The SPWLA52nd Annual Logging Symposium: Colorado (2011).

[10] Collet TS (2011). Downhole well log and core montages from the Mount Elbert Gas Hydrate Stratigraphic Test Well, Alaska North Slope. Marine and Petroleum Geology..

[11] Collett TS (2012). Gulf of Mexico Gas Hydrate Joint Industry Project Leg II logging-while-drilling data acquisition and analysis. Marine and Petroleum Geology..

[12] Majumdar U (2017). Semi-quantitative gas hydrate assessment from petroleum industry well logs in the northern Gulf of Mexico. Marine and Petroleum Geology..

[13] Ning FL (2013). Well logging assessment of natural gas hydrate reservoirs and relevant influential factors. Acta Petrolei Sinica..

[14] Lin ZZ (2013). Physical Analysis with the Logging data on Natural Gas Hydrate in Qilian Mountain permafrost area. Geophysical and Geochemical Exploration..

[15] Max MD, Johnson AH (2014). Hydrate petroleum system approach to natural gas hydrate exploration. Petroleum Geoscience..

[16] Sha ZB (2015). A seepage gas hydrate system in northern South China Sea: Seismic and well log interpretations. Marine Geology..

[17] Miyakawa A (2014). Gas hydrate saturation at Site C0002, IODP Expeditions 314 and 315, in the Kumano Basin, Nankai trough. Island Arc..

[18] Wang XJ (2013). Anomlous wireline logging data caused by gas hydrate dissociation in the Shenhu area, northern slope of South China Sea. Chinese Journal of Geophysics..

[19] Wang XJ (2011). Elevated gas hydrate saturation within silt and silty clay sedimentsin the Shenhu area, South China Sea. Journal of Geophysical Research..

[20] Zhao J (2016). Review on Logging Technology Evaluation Methods of Nature Gas Hydrate. Well Logging Technology..

[21] Lee MW, Waite WF (2008). Estimating pore-space gas hydrate saturations from well log acoustic data. Geochemistry, Geophysics, Geosystems..

[22] Lee MW, Collett TS (2009). Gas hydrate saturations estimated from fractured reservoir at SiteNGHP-01-10, Krishna-Godavari Basin. India. Journal of Geophysical Research..

[23] Lee MW, Collett TS (2012). Pore- and fracture-filling gas hydrate reservoirs in the Gulf of Mexico Gas Hydrate Joint Industry Project Leg II Green Canyon 955 H well. Marine and Petroleum Geology..

[24] Lu JA (2008). Well Logging Evaluation of Gas Hydrates in Shenhu Area, South China Sea. Geoscience..

[25] Daigle H, Cook A, Malinverno A (2015). Permeability and porosity of hydrate-bearing sediments in the northern Gulf of Mexico. Marine and Petroleum Geology..

[26] Zhou, Y. Research on Logging Interpretation Method of Gas Hydrates. Jilin University: China Changchun (2010).

[27] Ma, L. Numerical Simulation of Saturation Parameters in Gas Hydrate-bearing Reservoirs. Jilin University: China Changchun (2014).

[28] Jia JH, Takeshi T, Toshifumi M (2017). Gas hydrate saturation and distribution in the Kumano Forearc Basin of the Nankai Trough. Exploration Geophysics..

[29] Pang, S. J. Relationship between tectonic, sedimentation characteristics and distribution of gas hydrate in Muli Coalfield of Qilian Mountain. China University of Geosciences: China Beijing (2012).

[30] Wen HJ, Lu J, Shang LJ (2006). A Sequence Stratigraphic Discussion of the Jurassic Coal Measures in the Juhugeng Coalmine Area in Qinghai Province. Coal Geology of China..

[31] Lu ZQ (2015). Study on the accumulation pattern for permafrost-associated gas hydrate in sanlutian of Muli, Qinghai. Geoscience..

[32] Wang ZW (2003). Logging identification and evaluation methods for gas hydrate. Marine Geology & Quaternary Geology..

[33] Lee MW (1993). Method of estimating the amount of *in situ* gas hydrate in deep marine sediments. Marine and Petroleum Geology..

